# Supplementary material for: fCUT&Tag‐Seq: An optimized CUT&Tag‐based method for high‐resolution profiling of histone modifications and chromatin‐binding proteins in fungi
Source: mLife. 2026 Mar 23;5(2):239–53. doi: 10.1002/mlf2.70060 (PMC13131329; doi:10.1002/mlf2.70060)
Supplement: Supplementary file 1 — Fig.S1 V. dahliae V592 genome assembly presentation. (A) Output quality of sequencing data. Sample: sample name; SeqNum: number of sequences; SumBase: total number of databases; N50Len: N50 length of sequencing data; N90Len: length of sequencing data N90; MeanLen: mean length of sequencing sequence; MaxLen: maximum length of sequencing sequence. (B) Assembly result statistic. Length: length of the sequence after concatenation; GC (%): GC content of the concatenated sequence. (C) Coding gene prediction results. (D) CIRCOS plot of the genome. Fig.S2 Optimization and comparative evaluation of the fCUT&Tag‐Seq protocol for V. dahliae. (A). Genome browser view of H3K9me3 distribution in wild‐type and VdΔkmt1 strains using nucleus, spores, or protoplast. (B) Comparison of the state of protoplasts extracted by different methods. The upper panel shows that no spheroplasting state was observed, extracted by zymolyase described in the CUT&RUN approach for Candida albicans and the CUT&Tag method for Saccharomyces pombe, and the lower panel shows the protoplast state using the optimized fCUT&Tag‐Seq technique. The images were taken using a confocal microscope (Leica TCS SP8). Scale bar: 25 µm. (C) Genome browser view of H3K9me3 distribution in wild‐type and VdΔkmt1 strains using different cell numbers. (D) Genome browser view of H3K9me3 distribution in wild‐type and VdΔkmt1 strains using different sequencing volumes. (E) Genome browser view of H3K9me3 distribution in wild‐type and VdΔkmt1 strains using fCUT&Tag‐Seq and ChIP‐Seq, respectively. The Y‐axis values in ChIP‐seq tracks represent normalized read coverage depth (in reads per million, RPM), calculated by scaling raw read counts to 10⁶. Fig.S3 Genome‐wide profiling of nonspecific IgG signals and histone modification patterns in V. dahliae using fCUT&Tag‐Seq. (A) Genome browser view of the H3K9me3, H3K27me3, and nonspecific IgG antibody signals across representative genomic regions in the wild‐type strain. Data were presen [file MLF2-5-239-s001.pdf]

A

Sample	SeqNum	SumBase	N50Len	N90Len	MeanLen	MaxLen
Verticillium dahliae V592	156947206	40711754401	452	136	64.849	9518

B

Chr	Chr1	Chr2	Chr3	Chr4	Chr5	Chr6	Chr7	Chr8
Length	4416504	7834363	5191406	4182892	3710032	3337687	3268461	3754928
GC%	52.88	54.63	51.5	52.89	53.32	53.11	52.31	55.27

C

Sample	Gene Number	CDS Averange length	CDS Averange GC(%)
Verticillium dahliae V592	8332	1435.8863	59.55

D

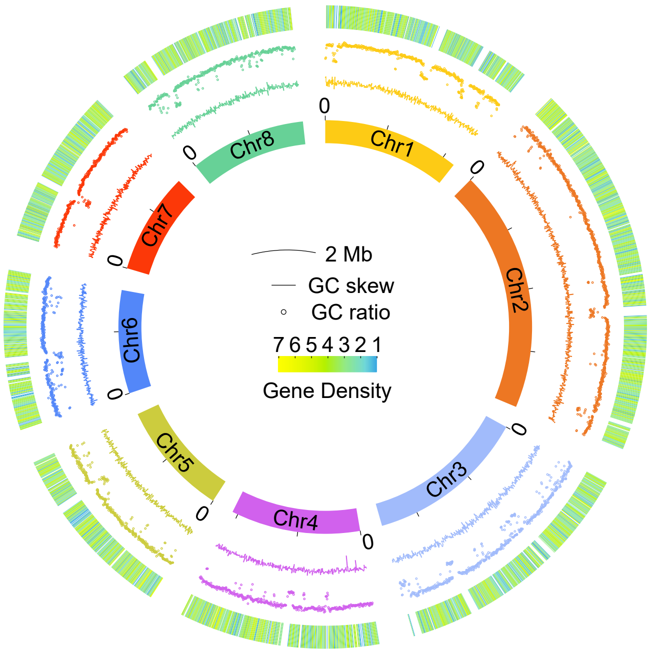

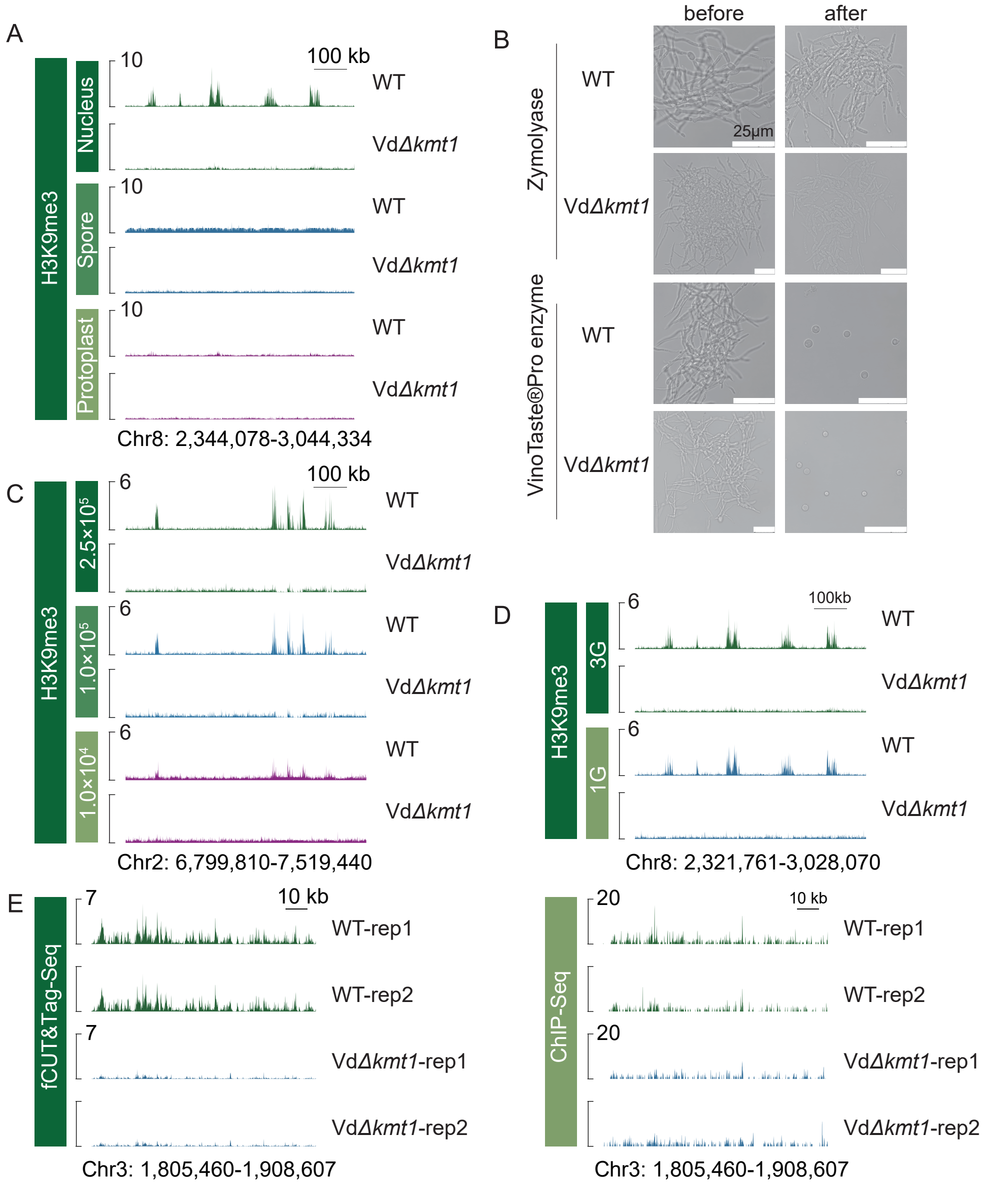

A
H3K9me3
Nucleus
Spore
Protoplast
10
100 kb
WT
VdΔkmt1
Chr8: 2,344,078-3,044,334
B
before
after
Zymolyase
VinoTaste®Pro enzyme
WT
VdΔkmt1
25μm
C
H3K9me3
2.5×10⁵
1.0×10⁵
1.0×10⁴
6
100 kb
Chr2: 6,799,810-7,519,440
D
H3K9me3
3G
1G
6
100kb
Chr8: 2,321,761-3,028,070
E
fCUT&Tag-Seq
ChIP-Seq
7
20
10 kb
WT-rep1
WT-rep2
VdΔkmt1-rep1
VdΔkmt1-rep2
Chr3: 1,805,460-1,908,607

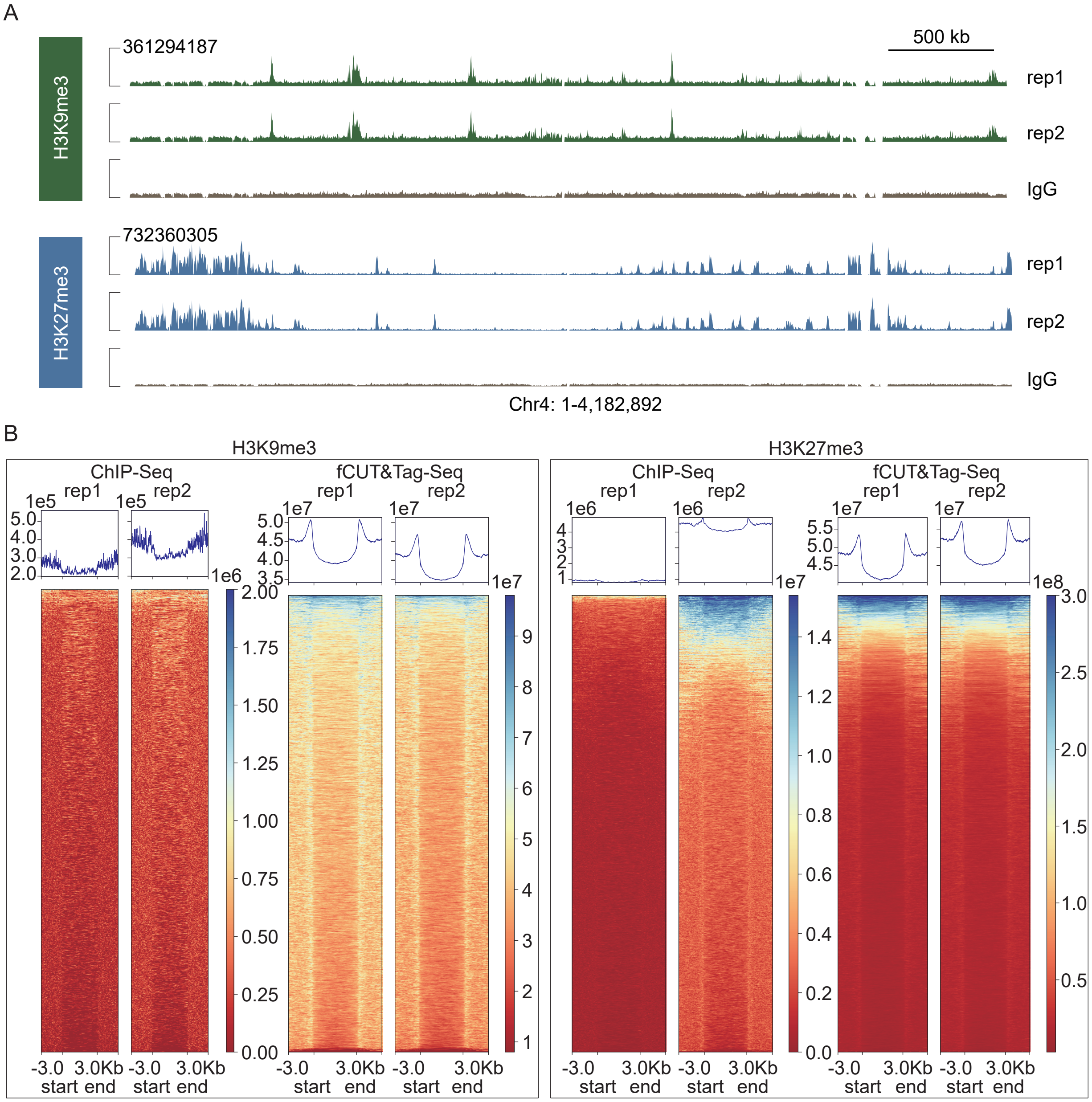
A
500 kb
361294187
H3K9me3
rep1
rep2
IgG
732360305
H3K27me3
rep1
rep2
IgG
Chr4: 1-4,182,892
B
H3K9me3
ChIP-Seq
fCUT&Tag-Seq
H3K27me3
ChIP-Seq
fCUT&Tag-Seq
rep1
rep2
start
end
-3.0
3.0Kb

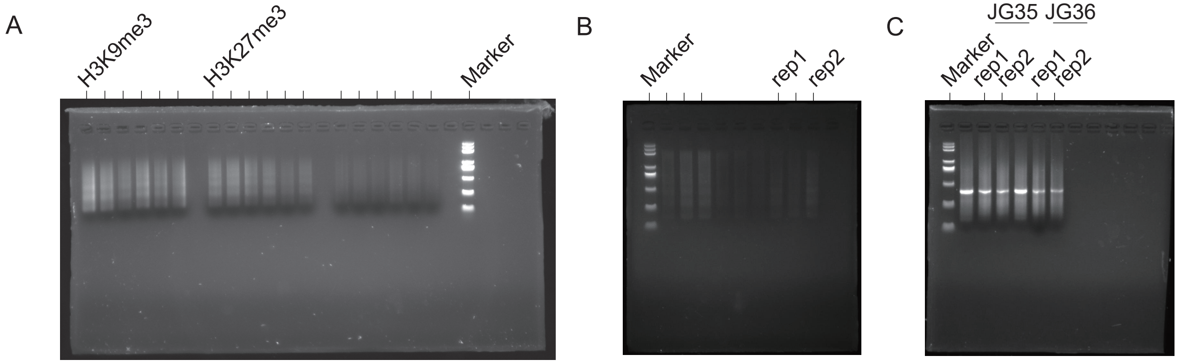
A
H3K9me3
H3K27me3
Marker
B
Marker
rep1
rep2
C
JG35
JG36
Marker
rep1
rep2
rep1
rep2

Table S1: Primers used in this study.

Primers	Sequences (5'→3')	Application
VdKMT1-A	AATTCGAGCTCGCTGAGGGTTTAATTAACAACATTACGCAGCTCTACGAGT	Vd*Δkmt1* construct
VdKMT1-a	TCGATGGGCCCGCTGAGGACTTAATTAAGTTGACTGATCTGTAAATAAG	
VdKMT1-d	CCCCGACTAGTGCTGAGGCATTAATTAACGAGGCACCGGATGGGAGGG	
VdKMT1-B	TCACGAAGCTTGCTGAGGTCTTAATTAAGTCGTGATGGTGCCTGTGTGGAG	
Hpt-F	TCTCCTTGCATGCACCATTCCTTG	Detection of Vd*Δkmt1*
HPT-R	GCAGCTATTTACCCGCAGGA	
VdKMT1-V1	CTCAGGGCAACATTGGAGGT	
VdKMT1-V2	CAAGACAGCAAGCACGAGAAG	
VdEZH2-A	TTCGAGCTCGCTGAGGGTTTAATTAAATTCAAGGGTTTGGATCGG	Vd*Δezh2* construct
VdEZH2-a	GATGGGCCCGCTGAGGACTTAATTAATGCGAATCTGCTTGTGAGG	
VdEZH2-d	CCGACTAGTGCTGAGGCATTAATTAAGGATTTCCTGCCATGCAC	
VdEZH2-B	ACGAAGCTTGCTGAGGTCTTAATTAAAGGGTAAGGCAGGGACCG	
Hpt-F	TCTCCTTGCATGCACCATTCCTTG	Detection of Vd*Δezh2*
HPT-R	GCAGCTATTTACCCGCAGGA	
VdEZH2-V1	GGCATGGTGTGTAATTGAAA	
VdEZH2-V2	TGTGACCTCGCCGCTATTG	
SsGcn5-tg-A	AGATGACCGTGAACCACTCG	SsGcn5-Flag construct
SsGcn5-tg-a	CCGTCATGGTCTTTGTAGTCCAAGGAACTCTGCACCTTCG	
SsGcn5-d	GTCCGCAATGTGTTATTAAGTGGTCCCAAGGTTGTCTTG	
SsGcn5-B	GGTGTAGATGGCGAGGAGAT	
HPT-LB-F	GACTACAAAGACCATGACGG	
HPT-LB-R	GGTCAAGACCAATGCGGAGC	
HPT-RB-F	GCAAGACCTGCCTGAAACCG	
HPT-RB-R	CTTAATAACACATTGCGGAC	
SsGcn5-C (V1)	TCCGGGCCACACTCTTC	Detection of SsGcn5-Flag
SsGcn5-D (V2)	ATGGCAAGGTTGAGGCTGT	
